# Features of the Duckweed *Lemna* That Support Rapid Growth under Extremes of Light Intensity

**DOI:** 10.3390/cells10061481

**Published:** 2021-06-12

**Authors:** Jared J. Stewart, William W. Adams, Marina López-Pozo, Naiara Doherty Garcia, Maureen McNamara, Christine M. Escobar, Barbara Demmig-Adams

**Affiliations:** 1 Department of Ecology and Evolutionary Biology, University of Colorado, Boulder, CO 80309, USA; william.adams@colorado.edu (W.W.A.III); marina.lopezpozo@colorado.edu (M.L.-P.); naiara.garcia@colorado.edu (N.D.G.); maureen.mcnamara@colorado.edu (M.M.); 2 Department of Aerospace Engineering Sciences, University of Colorado, Boulder, CO 80309, USA; chris@spacelabtech.com; 3 Space Lab Technologies, LLC, Boulder, CO 80309, USA

**Keywords:** antioxidants, carotenoids, chlorophyll fluorescence, photochemical efficiency, protein, tocopherol, xanthophyll cycle, zeaxanthin

## Abstract

This study addresses the unique functional features of duckweed via comparison of *Lemna gibba* grown under controlled conditions of 50 versus 1000 µmol photons m^−2^ s^−1^ and of a *L. minor* population in a local pond with a nearby population of the biennial weed *Malva neglecta*. Principal component analysis of foliar pigment composition revealed that *Malva* was similar to fast-growing annuals, while *Lemna* was similar to slow-growing evergreens. Overall, *Lemna* exhibited traits reminiscent of those of its close relatives in the family Araceae, with a remarkable ability to acclimate to both deep shade and full sunlight. Specific features contributing to duckweed’s shade tolerance included a foliar pigment composition indicative of large peripheral light-harvesting complexes. Conversely, features contributing to duckweed’s tolerance of high light included the ability to convert a large fraction of the xanthophyll cycle pool to zeaxanthin and dissipate a large fraction of absorbed light non-photochemically. Overall, duckweed exhibited a combination of traits of fast-growing annuals and slow-growing evergreens with foliar pigment features that represented an exaggerated version of that of terrestrial perennials combined with an unusually high growth rate. Duckweed’s ability to thrive under a wide range of light intensities can support success in a dynamic light environment with periodic cycles of rapid expansion.

## 1. Introduction

Small, floating plant species in the duckweed family (Lemnaceae) possess attractive nutritional features as they accumulate large quantities of high-quality protein (with all essential amino acids for humans) throughout the plant [1]. Furthermore, our group has highlighted the exceptional ability of *Lemna gibba* to accumulate high levels of the carotenoid zeaxanthin under conditions when the plant is growing rapidly [2,3]. Zeaxanthin (and its close isomer lutein) is an essential human micronutrient required to support brain function and fight systemic inflammation [4]. Duckweed also has potential uses in sustainable agricultural systems as food for humans, feed for animals (via conversion of wastewater to feed [5,6]), for other valuable products [7], or to improve nitrogen-use efficiency and yield of crops like rice [8]. Here, we present further insight into how *L. gibba* is able to combine fast growth across a range of environments with high nutritional content including pronounced zeaxanthin accumulation as well as other essential nutrients for humans or livestock.

We previously reported a notable ability of *L. gibba* to maintain uniformly high growth rates, paired with profound modulation of photoprotection, over a range of growth photon flux densities (PFDs) from 100 to 700 µmol m^−2^ s^−1^ of continuous light [2]. Plants grown under higher PFDs exhibited higher levels of the interconvertible xanthophyll cycle carotenoids (violaxanthin, antheraxanthin, and zeaxanthin) and pronounced conversion to zeaxanthin that dissipates potentially harmful excess absorbed light [3,9,10]. In the present study, we further broadened the range of growth PFDs to test whether duckweed’s phenotypic plasticity with respect to photoprotective capacity and maintenance of a high growth rate may extend to even more extreme growth PFDs. We compared features of *L. gibba* grown under very low (50 µmol photons m^−2^ s^−1^) or very high (1000 µmol photons m^−2^ s^−1^) intensity of continuous light under otherwise common, controlled conditions. Continuous exposure (24 h per day) to the high growth PFD represented a greater total daily photon flux than that on the longest, brightest day on Earth. Beyond extending the range of PFDs versus the previous study [2], additional parameters were characterized in the present study including the light-use efficiency of biomass production as well as the production of protein as a key macronutrient and α-tocopherol (vitamin E) as an additional micronutrient. Moreover, CO_2_-saturated photosynthetic capacity was characterized under both saturating light and the respective contrasting growth PFDs, and photosynthesis as well as protein and all micronutrients were expressed on multiple reference bases (per frond area, biomass, and chlorophyll [Chl] content) for a fuller evaluation of both plant function and nutritional quality for the consumer.

Furthermore, the present study tested the hypothesis that the combination of exceptionally rapid growth with a remarkable ability to grow under a wide range of light intensities in duckweed may be associated with pigment patterns not seen in other fast-growing species. In particular, prior studies of leaf pigment composition in slow-growing evergreens or perennials versus fast-growing annual species often reported an inverse relationship between growth rate and accumulation of photoprotective pigments (for a review, see [3]). The present study compared a population of *Lemna minor* in an open outdoor pond with a nearby population of the fast-growing terrestrial biennial weed *Malva neglecta* that was previously shown to exhibit a pigment composition and photoprotective capacity similar to that of fast-growing annual crop species [11]. Foliar pigment composition of *M. neglecta* and *L. minor* growing in full sun outdoors as well as that of *L. gibba* grown in low versus high PFD under controlled conditions were compared via principal component analysis to foliar pigment data for other species groups (including annuals as well as evergreens and other perennials).

## 2. Materials and Methods

### 2.1. Plant Material and Growth Conditions

#### 2.1.1. Controlled Conditions

Cultures of *Lemna gibba* L. 7741 (G3) obtained from Rutgers Duckweed Stock Cooperative (http://ruduckweed.org; accessed on 10 June 2021) were grown under controlled conditions in Conviron PGR15 and E15 growth chambers (Controlled Environments Ltd., Winnipeg, MB, Canada). Plants were grown in 150 × 75 mm PYREX Crystallizing Dishes (Corning Inc., Corning, NY, USA) that contained 1000 mL of freshly prepared Schenk and Hildebrandt medium (bioWORLD, Dublin, OH, USA [12]) at a concentration of 1.6 g L^−1^ (pH adjusted to 5.5 via 1% [*w*/*v*] KOH). Plants were cultivated continuously under 50 μmol photons m^−2^ s^−1^ (provided by F72T12/CW/HO fluorescent bulbs [Philips, Somerset, NJ, USA] and 100 W, 130 V incandescent bulbs [EiKO, Shawnee, KS, USA]) at 25 °C, and a subset of plants from each dish were transferred to clean dishes containing freshly prepared media at least once per week.

Plants from the cultures under 50 μmol photons m^−2^ s^−1^ were acclimated to 1000 μmol photons m^−2^ s^−1^ (provided by M47/E 1000 W metal halide bulbs; Philips, Somerset, NJ, USA) following the procedure described by Stewart et al. [2]. A subset of plants from the stock cultures (approximately 20 fronds per dish) were transferred to 200 μmol photons m^−2^ s^−1^ (supplied by C503C-WAN white LEDs; CREE Inc., Durham, NC, USA) for three days and then transferred to 1000 μmol photons m^−2^ s^−1^ for three days. After each of these three-day acclimation phases, a subset of plants (approximately 20 fronds per dish) that had developed under the prevailing light intensities were transferred to clean dishes with freshly prepared and filtered media. This process ensured that the characterized plant material developed under 1000 μmol photons m^−2^ s^−1^ and had not developed under a lower PFD and then been transferred to 1000 μmol photons m^−2^ s^–1^. Following this six-day acclimation process, plants were characterized over the course of four days while growing under 1000 μmol photons m^−2^ s^−1^ and a media temperature of 25 °C.

#### 2.1.2. Field Conditions

Populations of *Lemna minor* L. and *Malva neglecta* Wallr. plants (identification based on morphology and geographic distribution) growing naturally in Superior, CO, USA (39°56′28″ N, 105°09′02″ W) were characterized. *Malva neglecta* is a relatively fast-growing herbaceous biennial species that remains photosynthetically active throughout the year in this area (see [13]). The *L. minor* plants were growing in a slow-moving section of a small stream, and the *M. neglecta* plants were growing on a south-facing slope immediately north of the stream. Both locations received direct, midday sunlight. Samples for pigment analysis were collected during exposure to full sun (maximal PFD of 1600 µmol m^−2^ s^−1^) prior to solar noon on 17 May 2019. Samples were imaged (for quantification of frond/leaf area via ImageJ [14]) and then submerged and stored in liquid nitrogen at the field site. The four characterized samples of *L. minor* each consisted of multiple fronds from multiple plants (i.e., multiple biological replicates per sample), whereas the four characterized samples from *M. neglecta* each consisted of one leaf segment from four separate plants (i.e., four biological replicates).

### 2.2. Growth Metrics

Under controlled conditions, the dishes containing *L. gibba* plants were imaged from directly overhead once per day during the four-day period of characterization, and the frond area was quantified from these images using MATLAB Image Processing Toolbox (MathWorks, Natick, MA, USA) as previously described [2]. Dry mass of whole plants (i.e., fronds with intact roots) and only fronds (i.e., fronds with excised roots) was measured from samples that had been dried at 70 °C for seven days. Prior to drying, each sample was imaged from directly overhead, and the frond area was determined from these images using ImageJ [14].

Relative growth rate was calculated by dividing the difference in natural logarithm–transformed frond areas at the end and the beginning of the four-day experimental period by the time elapsed between the two measurements. Doubling time was calculated as the natural logarithm of 2 divided by relative growth rate. Light-use efficiency of frond area production was calculated as the accumulated frond area (i.e., the difference between the final and initial frond areas) divided by the number of incident photons on the frond surface (calculated as described in detail by Stewart et al. [2]). Light-use efficiency of biomass production was estimated as the accumulated biomass (i.e., product of accumulated frond area [m^2^] and whole-plant dry mass per unit frond area [g m^−2^]) divided by the number of available photons during this time period.

### 2.3. Photosynthesis and Respiration

Rates of photosynthetic oxygen evolution were determined as described by Stewart et al. [2] with saturating CO_2_ (5% CO_2_, 21% O_2_, balance N_2_) using leaf disc oxygen electrodes (Hansatech Instruments Ltd., Norfolk, United Kingdom; see [15]) and a circulating water bath set to 25 °C. Fronds from *L. gibba* plants grown under 50 µmol photons m^−2^ s^−1^ and 1000 µmol photons m^−2^ s^−1^ were assayed under their respective growth PFDs as well as a saturating PFD of 1500 µmol photons m^−2^ s^−1^. Respiration rates were determined following measurements of photosynthesis as the rate of oxygen consumption in darkness.

Photochemical and photoprotective processes were assessed via measurements of chlorophyll fluorescence with a PAM-101 chlorophyll fluorometer (Walz, Effeltrich, Germany) following the procedures described in detail by Stewart et al. [2] and using calculations described by Demmig-Adams et al. [16]. To ascertain the maximal level of fluorescence (F_m_ or F_m_′) in high-light flashes, two or three flashes were given in rapid succession [17], which revealed that the maximal attainable level was reached during the first flash under all conditions used here.

### 2.4. Protein and Starch

Total protein content was quantified spectrophotometrically with the Total Protein Kit, Micro Lowry, Peterson’s Modification (Sigma-Aldrich, Saint Louis, MO, USA), which follows a modified version [18] of the procedure described by Lowry et al. [19]. Whole plants with approximately three fronds per dish, which had been imaged and immediately frozen in liquid nitrogen, were homogenized via mortar and pestle, combined with 1 mL of water, vortexed, and centrifuged for 10 min at 10,000 rpm. The resulting supernatant was decanted, combined with 0.1 mL of deoxycholate, vortexed, and maintained at room temperature for 10 min. Subsequently, 0.1 mL of trichloroacetic acid was added, and this solution was vortexed and centrifuged for 10 min at 10,000 rpm. The resulting pellet was re-suspended in 1 mL of Lowry reagent, transferred to cuvettes, and then mixed with an additional 1 mL of water that was used to rinse the microcentrifuge tube. After 20 min of incubation at room temperature, 0.5 mL of the Folin–Ciocalteu phenol reagent was added, and this solution was mixed via pipette and incubated at room temperature for 30 min. Absorbance at 660 nm was determined with a Beckman DU 640 Spectrophotometer (Beckman Instruments, Inc., Fullterton, CA, USA) and these values were converted to protein levels (in µg mL^−1^) using a standard calibration curve based on a gradient of bovine serum albumin.

The abundance of starch in *L. gibba* plants was detected qualitatively with a diluted iodine-potassium iodide solution (Lugol’s solution; Sigma-Aldrich, St. Louis, MO, USA). Plants were cleared in 70% (*v*/*v*) ethanol, stained for 5 min, and then immediately mounted and imaged with a high-resolution scanner (Perfection 3200 Photo; Epson America, Inc., Long Beach, CA, USA).

### 2.5. Chlorophyll, Carotenoid, and α-Tocopherol Levels

Chlorophylls *a* & *b*, lutein, zeaxanthin (Z), antheraxanthin (A), violaxanthin (V), neoxanthin, β-carotene, and α-tocopherol levels were quantified via high-performance liquid chromatography as previously described in detail [2,20]. Under controlled conditions, samples of approximately 10 fronds per dish were collected under the respective growth PFDs, imaged, and then frozen and stored in liquid nitrogen.

Pigment data from a previously conducted survey of multiple species in the same area by Demmig-Adams and Adams [21], which included *M. neglecta*, were used in the present study and divided into three groups: (i) shade-grown perennials; (ii) sun-grown perennials; and (iii) sun-grown annuals (for detail, see Table S1). To ensure the datasets were comparable, pigments were characterized from samples of *Vinca minor* plants growing in complete shade (shade-grown perennials) as well as plants growing exposed to full sun (sun-grown perennials) on the University of Colorado campus (Boulder, CO, USA) collected during the afternoon of 16 April 2019.

### 2.6. Statistical Analyses

Comparisons of means were preceded by Levene’s tests to assess the equality of variances. Comparison of two means were made with Student’s (equal variances) or Welch’s (unequal variances) *t*-tests, and comparisons of more than two means were made with one-way ANOVAs and post-hoc Tukey–Kramer HSD. Linear relationships between two variables were evaluated with Pearson correlations. Comparisons with multiple parameters were conducted with principal component analysis on correlations. All statistical analyses were made using JMP Pro 15.0.0 (SAS Institute Inc., Cary, NC, USA).

## 3. Results

### 3.1. Growth and Photosynthesis of Lemna gibba under Extremes in Growth PFD

**Figure 1:**
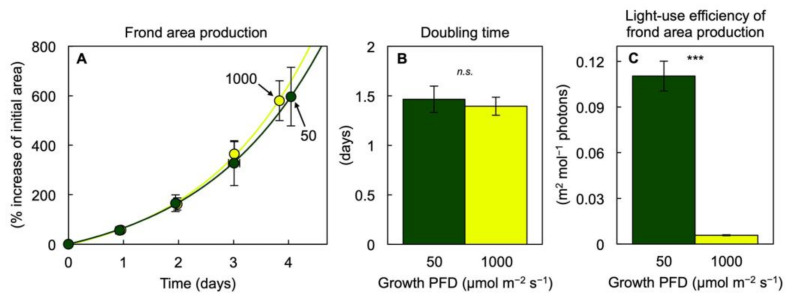
(**A**) Accumulation of frond area (% relative to frond area at the beginning of the experiment) over four days of growth, (**B**) average doubling time in frond area over this four-day period, and (**C**) light-use efficiency of frond area production (total frond area produced relative to incident PFD during the four-day growth period) in *Lemna gibba* plants under growth PFDs of 50 (green) or 1000 (yellow) µmol m^−2^ s^−1^. Mean values ± standard deviations; *n* = 7 for 50 µmol photons m^−2^ s^−1^; *n* = 3 for 1000 µmol photons m^−2^ s^−1^. A significant difference between the growth PFDs is denoted by asterisks in (**C**); *** = *p* < 0.001; *n.s.* = not significantly different.

Despite vastly different light availability (a 20-fold difference between 50 versus 1000 µmol photons m^−2^ s^−1^ of continuous light) during growth, a similar amount of duckweed frond area accumulated over time in the two controlled conditions (Figure 1A). This represented a similar frond area doubling time of just under 1.5 days for either of the two growth PFDs (Figure 1B), which also corresponded to similar average relative growth rates of 0.48 ± 0.04 and 0.50 ± 0.03 day^−1^ (average daily increase in natural logarithm–adjusted frond area over the four-day experimental phase) for plants grown under 50 and 1000 µmol photons m^−2^ s^−1^, respectively. As a consequence of the similar frond area accumulation under the two vastly different growth PFDs, the ratio of frond area produced per incident PFD, which can be considered the light-use efficiency of frond area production, was dramatically greater (1733%) in fronds grown under 50 versus 1000 µmol photons m^−2^ s^−1^ (Figure 1C), in other words, almost proportional to the 20-fold (or 2000%) difference in incident PFD.

**Figure 2:**
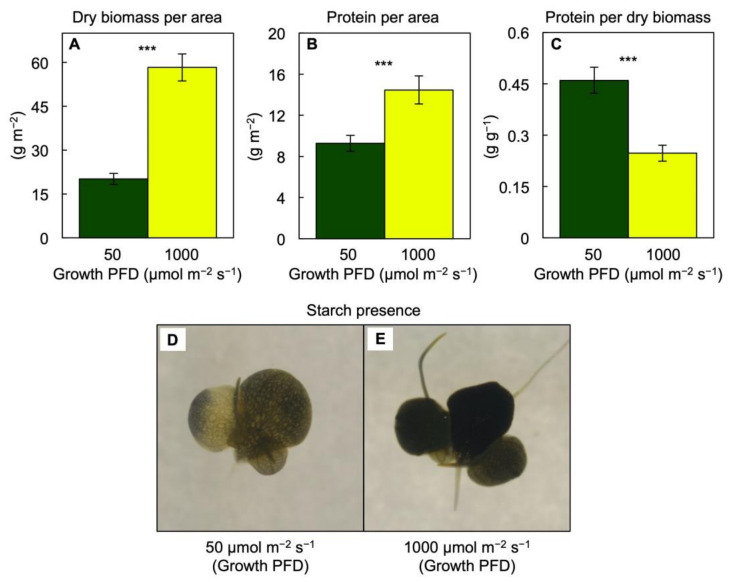
(**A**) Dry biomass per frond area, (**B**) protein per frond area, (**C**) protein per dry biomass, and (**D**,**E**) presence of starch (detected via diluted iodine-potassium iodide solution) in *Lemna gibba* plants grown under 50 (green) or 1000 (yellow) µmol photons m^−2^ s^−1^. Mean values ± standard deviations; *n* = 4. Significant differences between the growth PFDs are denoted by asterisks; *** = *p* < 0.001. Dimensions of each image (**D**,**E**) are 1 cm × 1 cm.

A greater amount of dry biomass (Figure 2A) and protein (Figure 2B) was accumulated on a frond area basis in plants growing under the higher PFD, but the fraction of dry biomass (% biomass in g g^−1^) that consisted of protein (Figure 2C) was greater under the lower PFD, which resulted in a remarkable 46% of dry biomass consisting of protein under the low growth PFD. There was also evidence for greater starch accumulation (Figure 2D,E) under the higher PFD. Another contributing factor to the difference in total dry biomass in plants grown under 1000 versus 50 µmol photons m^−2^ s^−1^ was a difference in root dry biomass, which accounted for 16% versus 6%, respectively. As was the case for area production, light-use efficiency of total biomass production on an incident light basis was much greater in fronds grown under 50 versus 1000 µmol photons m^−2^ s^−1^, albeit at a less pronounced percentage (672%) than seen for frond area (Figure 1C) due to the fact that, unlike frond area accumulation, dry mass accumulation was almost 3× greater at the higher growth PFD (Figure 2A).

**Figure 3:**
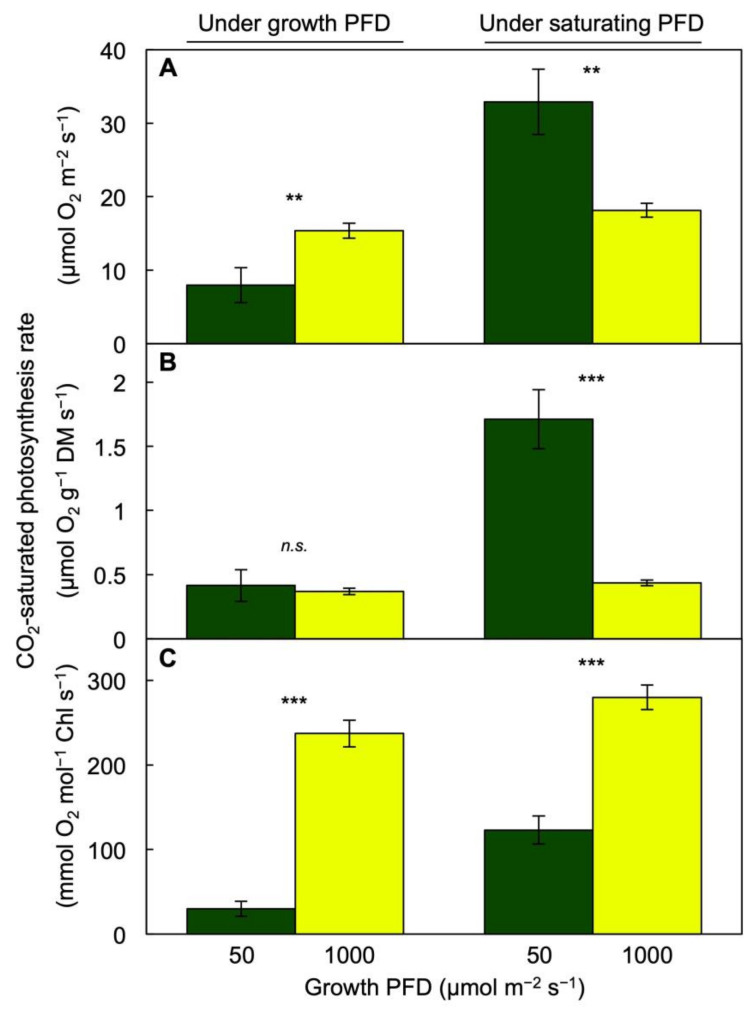
CO_2_-saturated rates of photosynthetic oxygen evolution of fronds on the bases of (**A**) area, (**B**) dry mass (DM), and (**C**) chlorophyll (Chl) *a* + *b* levels from *Lemna gibba* plants grown under PFDs of 50 (green) or 1000 (yellow) µmol m^−2^ s^−1^ and measured under the respective growth PFDs (left columns) as well as a common saturating PFD of 1500 µmol m^−2^ s^−1^ (right columns). Mean values ± standard deviations; *n* = 3. Significant differences between growth PFDs are denoted by asterisks; ** = *p* < 0.01, *** = *p* < 0.001, *n.s.* = not significantly different.

CO_2_-saturated photosynthesis rate was determined for plants grown under their respective growth PFDs of 50 and 1000 µmol m^−2^ s^−1^ as well as under a common saturating PFD of 1500 µmol m^−2^ s^−1^ (Figure 3). Furthermore, the resulting photosynthesis rates were expressed on three different reference bases (i.e., per frond area (Figure 3A), per frond dry biomass (Figure 3B), and per frond Chl content (Figure 3C)), revealing different trends. Photosynthesis on a frond area basis was higher in plants grown under the high PFD (Figure 3A) when measured under the respective PFDs (1000 versus 50 µmol m^−2^ s^−1^). Since fronds grown under high PFD accumulated a greater amount of biomass than fronds grown under the low PFD, the photosynthesis rate on a dry mass basis was similar in the fronds when measured at their respective growth PFDs (Figure 3B). Likewise, respiration rates were higher on an area basis (3.36 ± 0.39 versus 1.42 ± 0.82 µmol O_2_ m^−2^ s^−1^, *p* < 0.05) and similar on a dry mass basis (0.08 ± 0.01 versus 0.07 ± 0.04 µmol O_2_ g^−1^ s^−1^, *p* > 0.05) in fronds grown under 1000 versus 50 µmol photons m^−2^ s^−1^, respectively.

In contrast, light- and CO_2_-saturated photosynthetic capacity on an area basis (and even more so on a dry mass basis) measured under a saturating PFD of 1500 µmol m^−2^ s^−1^ was higher in the fronds grown under the low versus the high PFD (Figure 3A,B). Finally, photosynthesis rate on a Chl basis was much higher in the fronds grown under the high versus the low PFD when measured either under the respective growth PFDs or under saturating PFD (Figure 3C).

### 3.2. Pigment Composition, Light-Use Efficiency, and Photoprotection of Lemna gibba under Extremes in Growth PFD

**Figure 4:**
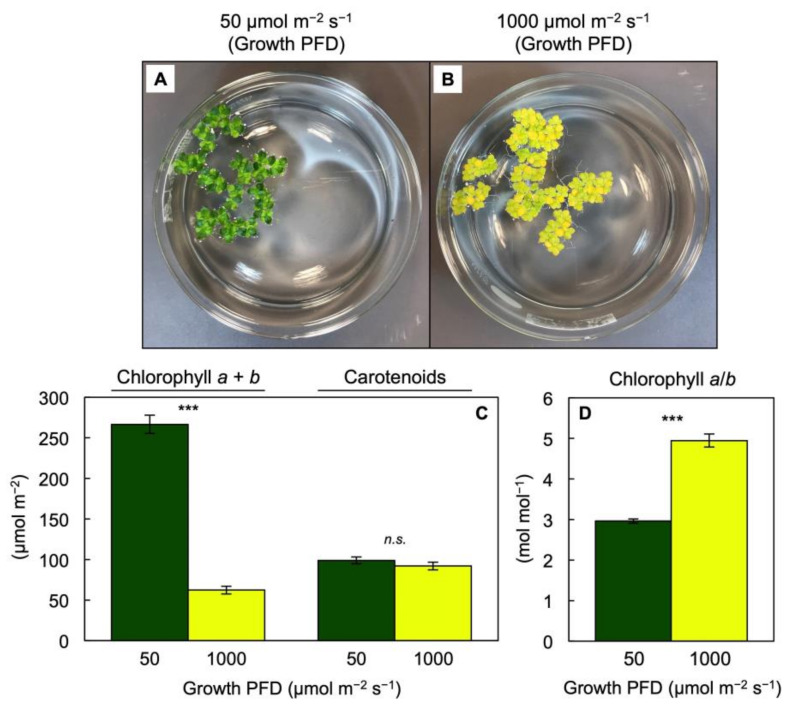
(**A**,**B**) Images of crystallizing dishes (1L volume) with representative cultures (starting from 20 fronds per dish) at the end of the four-day growth period, (**C**) chlorophyll *a* + *b* and carotenoid levels per frond area, and (**D**) the molar ratio of chlorophyll *a* to chlorophyll *b* in fronds of *Lemna gibba* grown under PFDs of 50 (green) or 1000 (yellow) µmol m^−2^ s^−1^. Mean values ± standard deviations; *n* = 3 or 4. Significant differences between growth PFDs are denoted by asterisks; *** = *p* < 0.001; *n.s.* = not significantly different.

The fronds grown under the low PFD were green (Figure 4A) whereas those grown under the high PFD were bright yellow (Figure 4B). This difference in visual appearance was associated with a much lower Chl content, but similar total carotenoid content, on a frond area basis in the fronds grown under 1000 versus 50 µmol photons m^−2^ s^−1^ (Figure 4C). This difference in Chl content is, furthermore, consistent with the much higher ratios of O_2_ evolution relative to Chl (Figure 3C) in the fronds grown under the high versus the low PFD (which contrasted with the similar or lower rates of O_2_ evolution on the frond area or dry mass bases, respectively). Despite the lower Chl content, the ratio of O_2_ evolution per absorbed photons would likely be considerably lower at the high versus the low PFD, but this ratio cannot be computed since the fraction of absorbed light available to photosynthesis (by way of chlorophyll as opposed to carotenoids, at least some of which may not harvest light for photosynthesis) in a yellow frond is unknown.

**Figure 5:**
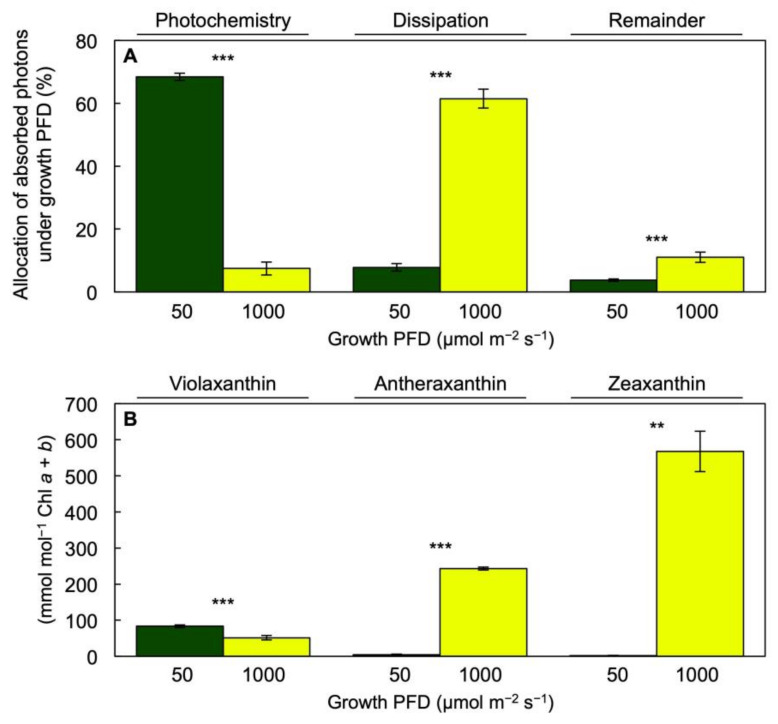
(**A**) Estimated percentages of absorbed light (under the respective growth PFDs) allocated to PSII photochemistry (F_v_′/F_m_′ × q_P_), dissipation (0.8 − F_v_′/F_m_′), and the fraction of excitation energy dissipated neither via photochemical or regulated non-photochemical routes (remainder; F_v_′/F_m_′ × [1 − q_P_]) and (**B**) levels of xanthophyll cycle pool constituents violaxanthin, antheraxanthin, and zeaxanthin relative to chlorophyll (Chl) *a* + *b* in fronds of *Lemna gibba* plants grown under PFDs of 50 (green) or 1000 (yellow) µmol m^−2^ s^−1^. The conversion state of the xanthophyll cycle to zeaxanthin and antheraxanthin in (**B**) should be compared to the level of dissipation in (**A**). Mean values ± standard deviations; *n* = 3 or 4. Significant differences between growth PFDs are denoted by asterisks; ** = *p* < 0.01, *** = *p* < 0.001.

Figure 5A shows an estimation (from chlorophyll fluorescence) of the allocation of absorbed light to PSII photochemistry (Photochemistry) and non-photochemical routes (Dissipation) as well as the fraction of excitation energy dissipated neither via photochemical or regulated non-photochemical routes (Remainder). The combination of photochemical and non-photochemical routes of excitation energy utilization or dissipation apparently was sufficient to prevent any major build-up of excitation energy.

**Figure 6:**
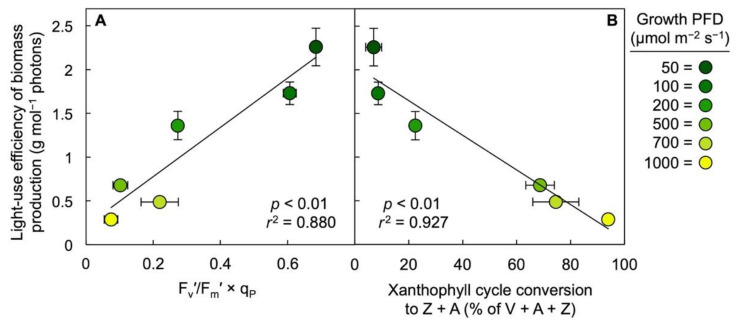
Correlations between the light-use efficiency of biomass production (total dry biomass produced per incident PFD during the four-day growth period) and (**A**) (F_m_′ − F)/F_m_′ = F_v_′/F_m_′ × q_P_ (as an estimate of the light-use efficiency of PSII photochemistry) or (**B**) the percent of the xanthophyll cycle pool converted to zeaxanthin (Z) and antheraxanthin (A) in *Lemna gibba* fronds grown under six PFDs ranging from 50 to 1000 µmol m^−2^ s^−1^. Mean values ± standard deviations, *n* = 3 or 4. Data for PFDs of 100 to 700 µmol m^−2^ s^−1^ were calculated from [2]. V = violaxanthin.

The much greater estimated dissipation of absorbed light via regulated non-photochemical routes (Figure 5A, Dissipation) in fronds grown under 1000 versus 50 µmol photons m^−2^ s^−1^ was mirrored by strong accumulation of the photoprotective xanthophylls zeaxanthin and antheraxanthin relative to Chl *a* + *b* (Figure 5B). Furthermore, Figure 6 shows that either the estimated fraction of absorbed light allocated to PSII photochemistry (Figure 6A) or the fraction of the interconvertible xanthophyll cycle pool (violaxanthin + antheraxanthin + zeaxanthin, V + A + Z) converted to the de-epoxidized forms zeaxanthin and antheraxanthin (Figure 6B) exhibited a significant positive relationship with the energy-use efficiency of total frond dry biomass production over a range of six growth PFDs from 50 to 1000 µmol m^−2^ s^−1^ [2]. For the energy-use efficiency of frond area production, significant correlations were likewise seen (not shown) with the estimated fraction of absorbed light allocated to PSII photochemistry (*p* = 0.008; *r*^2^ = 0.862) and xanthophyll cycle pool conversion state (*p* = 0.039; *r*^2^ = 0.694), respectively.

**Figure 7:**
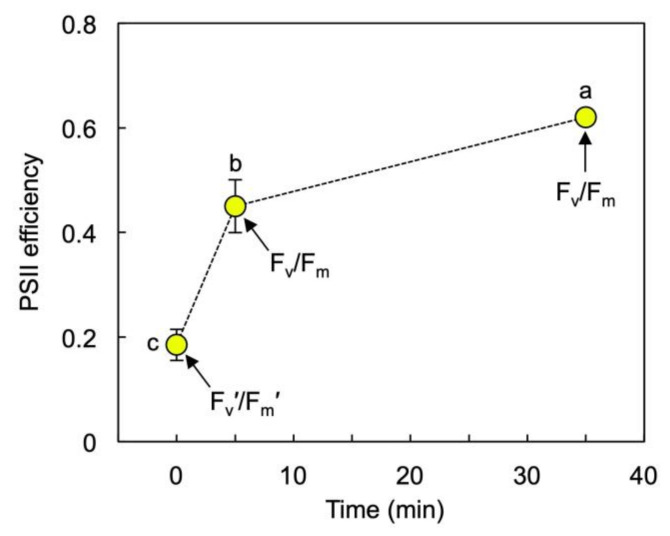
F_v_′/F_m_′ (as an estimate of intrinsic PSII efficiency during exposure to growth PFD) and F_v_/F_m_ (as an estimate of intrinsic PSII efficiency after 5 min darkness) for *L. gibba* fronds grown under 1000 µmol photons m^−2^ s^−1^. F_v_/F_m_ was determined at two time points (i.e., after 5 min of darkness upon removal from growth PFD and following a recovery period of 30 min under 10 µmol photons m^−2^ s^−1^ followed by 5 min darkness). Mean values ± standard deviations; *n* = 3. Significant differences between time points are denoted by different lower-case letters.

Figure 7 shows that the estimated fraction of absorbed light allocated to photosystem II photochemistry in fronds grown under 1000 µmol photons m^−2^ s^−1^ was lowered to less than 20% (F_v_′/F_m_′ < 0.2) under this growth PFD and rose rather quickly upon transfer of fronds to 10 µmol photons m^−2^ s^−1^ to over 60% (F_v_/F_m_ > 0.6) within 30 min. In contrast, there was only a small (albeit significant; *p* < 0.001) difference between the estimated fraction of absorbed light allocated to photosystem II photochemistry (not shown) under the growth PFD of 50 µmol photons m^−2^ s^−1^ (at 72.2 ± 1.2%) versus 30 min of recovery (78.1 ± 0.8%) in 10 µmol photons m^−2^ s^−1^.

A full characterization of pigment and α-tocopherol composition of *L. gibba* fronds grown under the extremes of 50 and 1000 µmol photons m^−2^ s^−1^ is presented in Table 1 and Table 2. Table 1 shows the levels of carotenoids and α-tocopherol on both frond area and dry biomass (DM) bases. Table 2 shows the ratios of carotenoids and α-tocopherol to Chl *a* + *b* or Chl *b* only as well as other ratios. While total carotenoid concentration under the high versus the low growth PFD was the same on a frond area basis (Figure 4), it was only about half on a DM basis (Table 1), but about 4× higher on a Chl *a* + *b* basis (Table 2). All individual carotenoids except zeaxanthin and antheraxanthin were present at lower concentrations on an area basis, and even lower on a DM basis (Table 1), while being enhanced relative to total Chl *a* + *b*, and even more so relative to Chl *b* alone, at the high versus the low growth PFD (Table 2). Zeaxanthin, antheraxanthin, and the total xanthophyll cycle pool (V + A + Z) were all greater on an area basis (Table 1) and relative to Chl *a* + *b* or Chl *b* alone (Figure 5B, Table 2). Zeaxanthin and antheraxanthin (Z + A), but not the total xanthophyll cycle pool, were also greater on a DM basis (Table 1). Moreover, higher ratios were seen at the high growth PFD for zeaxanthin or the total xanthophyll cycle pool to lutein, all xanthophylls to β-carotene, and the proportion of the total xanthophyll cycle pool that was converted to either zeaxanthin alone or the sum of zeaxanthin and antheraxanthin (Table 2). The only carotenoid present in the same ratio to Chl *a* + *b*, thus exhibiting a proportional decline as Chl *a* + *b* at the high growth PFD, was neoxanthin (Table 2). However, the neoxanthin level was not lowered as much as the Chl *b* level at the high growth PFD (Table 2). The only compound among those considered in Table 1 that was not significantly different in concentration on an area basis between the two growth PFDs was α-tocopherol, which resulted in a strong increase in the ratio of α-tocopherol to Chl *a* + *b* (Table 2). α-Tocopherol concentration was about half per DM at the high versus the low growth PFD (Table 1).

### 3.3. Unique Pigment Patterns in Lemna Compared to Other Species

**Figure 8:**
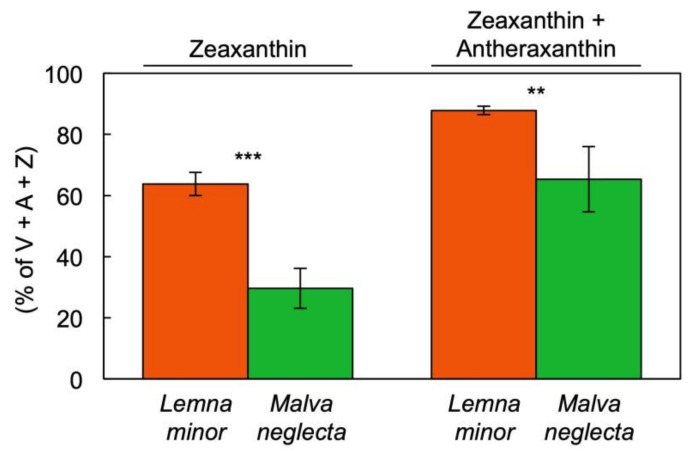
Conversion state of the xanthophyll cycle pool to zeaxanthin (Z) or zeaxanthin + antheraxanthin (A) under full sun (1600 µmol photons m^−2^ s^−1^) in fronds of *Lemna minor* (orange) and leaves of *Malva neglecta* (green) growing naturally in Superior, CO, USA. Mean values ± standard deviations; *n* = 4. Significant differences are denoted by asterisks; ** = *p* < 0.01, *** = *p* < 0.001. V = violaxanthin.

**Figure 9:**
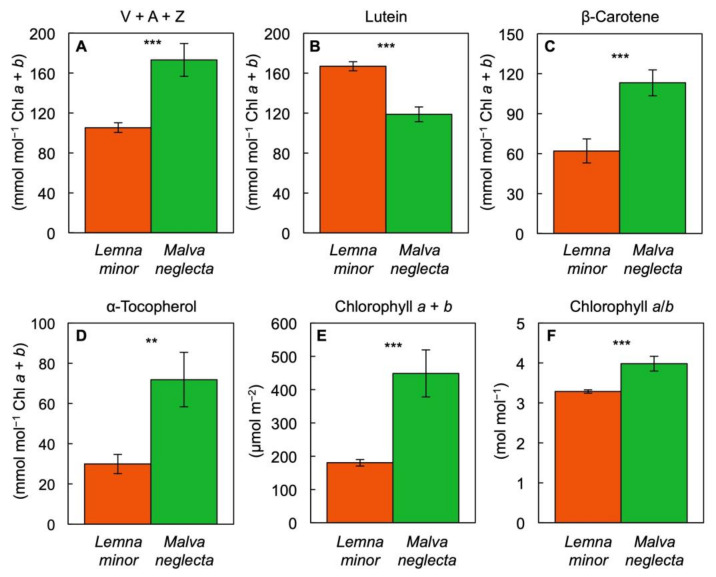
Levels of (**A**) the xanthophyll cycle pool constituents, violaxanthin, antheraxanthin, and zeaxanthin (V + A + Z), (**B**) lutein, (**C**) β-carotene, and (**D**) α-tocopherol per chlorophyll (Chl) *a* + *b*, (**E**) chlorophyll *a* + *b* levels per frond/leaf area, and (**F**) the molar ratio of chlorophylls *a* to *b* in fronds of *Lemna minor* (orange) and leaves of *Malva neglecta* (green) growing naturally in Superior, Colorado, USA. Mean values ± standard deviations; *n* = 4. Significant differences are denoted by asterisks; ** = *p* < 0.01, *** = *p* < 0.001.

Under exposure to full sunlight, fronds of *L. minor* floating on a local pond exhibited conversion of a greater percentage of the total pool of interconvertible xanthophylls (V + A + Z) to the photoprotective forms zeaxanthin or zeaxanthin + antheraxanthin compared to leaves of a nearby population of the weed *M. neglecta* (Figure 8). Whereas the conversion state to the photoprotective, de-epoxidized components was higher in *L. minor* (Figure 8), the total pool of the xanthophyll cycle relative to Chl *a* + *b* was smaller in *L. minor* compared to *M. neglecta* (Figure 9A).

Lutein was present at a greater level on a Chl basis in *L. minor* compared to *M. neglecta* (Figure 9B). In contrast, β-carotene per Chl (Figure 9C), α-tocopherol per Chl (Figure 9D), Chl *a* + *b* per area (Figure 9E), and the Chl *a*/*b* molar ratio (Figure 9F) were all lower in *L. minor* than in *M. neglecta*.

**Figure 10:**
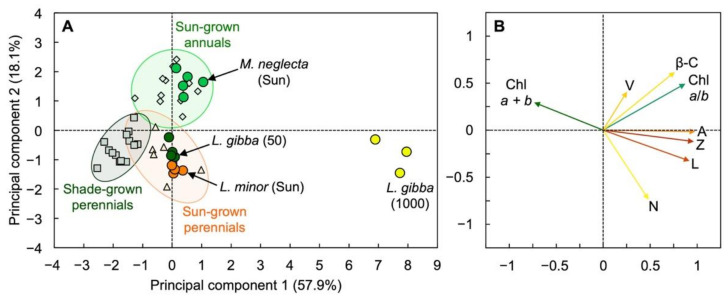
(**A**) Score and (**B**) loading plots of the first two principal components from principal component analysis on correlations of chlorophyll (Chl) *a* + *b* per leaf/frond area, Chl *a*/*b* molar ratio, and levels of violaxanthin (V), β-carotene (β-C), antheraxanthin (A), zeaxanthin (Z), lutein (L), and neoxanthin (N) per Chl *a* + *b* in fronds of *Lemna gibba* grown under PFDs of 50 (dark green circles) and 1000 (bright yellow circles) µmol m^−2^ s^−1^ in growth chambers, and fronds of sun-grown *Lemna minor* (bright orange circles), leaves of sun-grown *Malva neglecta* (bright green circles), and a variety of sun-grown annuals (light green diamonds), shade-grown perennials (gray squares), and sun-grown perennials (light orange triangles). For details, see Tables S1–S3 and [21].

The herbaceous weed *M. neglecta* (a biennial) fell into a cluster comprised of fast-growing annual species that accumulated rather modest amounts of zeaxanthin in full sun, while *L. minor* fell into a cluster of slow-growing perennial species (Figure 10A) using principal component analysis based on foliar pigment composition (Figure 10B). *Lemna minor*’s pigment composition was thus similar to that of slow-growing perennials, which accumulated large amounts of zeaxanthin and lutein in full sun. Remarkably, even *L. gibba* grown under 50 µmol photons m^−2^ s^−1^ fell into this cluster (Figure 10A), despite the fact that it did not accumulate zeaxanthin under this low growth PFD (Figure 5B). The yellow fronds of *L. gibba* grown under 1000 µmol photons m^−2^ s^−1^ that maintained carotenoids while strongly lowering Chl content (Figure 4C) fell far to the right of all three clusters (Figure 10A).

## 4. Discussion

The results of this study extend the conclusions reported in [2] of a notable ability of the duckweed *L. gibba* to grow rapidly across a range of growth PFDs from 100 to 700 µmol m^−2^ s^−1^. The present study extended this growth PFD range to include an even lower intensity of 50 µmol photons m^−2^ s^−1^ and an even higher intensity of 1000 µmol photons m^−2^ s^−1^ and documented the same high growth rate under the latter two extremes. This ability of duckweed to thrive under a wide range of light intensities makes sense in the context of duckweed ecology. Duckweed thrives in ponds where light environments can range from deep shade (at the pond’s edge where emergent macrophytes and/or overhanging willows, etc., may provide considerable shade) to full sun in the middle of an open pond, with rapid cycles of vegetative expansion (e.g., during spring upon activation of overwintering turions, after a pond is disrupted by inclement weather involving wind and/or recharge with a major precipitation event, etc.) experienced periodically.

### 4.1. Interspecies Comparison

Foliar pigment composition of a closely related species, *L*. *minor*, growing on an open pond in full sunlight was similar to those of slow-growing evergreens, in particular with respect to the high maximal conversion of the xanthophyll cycle pool to zeaxanthin and antheraxanthin at midday in sunny habitats (see, e.g., [21,22]). Duckweed thus exhibited a combination of the traits of fast-growing annuals and slow-growing evergreens with foliar pigment features reminiscent of evergreens but coupled with a growth rate that exceeds that of the fastest-growing terrestrial plants [23].

Duckweeds are fast-growing aquatic plants that are members of the monocotyledonous order Arales; duckweeds were previously considered a subfamily (Lemnoideae) of the Araceae, but are now grouped as their own family (Lemnaceae), which is closely related to the Araceae (for recent reviews of its taxonomy, see [24,25]). Terrestrial Araceae are common in shaded rainforest environments, possessing adaptations for high shade tolerance that make them suitable as house plants (e.g., genera such as *Alocasia*, *Dieffenbachia*, *Monstera*, *Philodendron* [26,27,28,29]). The foliar pigment composition, and its response to growth PFD, seen here in duckweed, was reminiscent of a member of the Araceae, *Monstera deliciosa* (subfamily Monsteroideae), which possesses a remarkable ability to acclimate to both deep shade and full sunlight [30]. This makes sense in the context of the ecology of some aroids as hemi-epiphytic vines that germinate in soil in deep shade on the rainforest floor, grow toward the darkest area (a tree trunk), climb into the forest canopy, lose connection to soil/ground, and thrive in dappled to full sunlight within the canopy [31,32,33].

On the other hand, their exceptionally high growth rates set duckweeds apart from terrestrial Araceae. The high growth rates of duckweed may be associated with genome reductions in these diminutive, floating plants that are associated with loss of controls placed on growth and stomatal conductance in terrestrial species [34,35]. Terrestrial species typically curb growth and stomatal opening under limiting water as a defense strategy [36]. This would appear less necessary for species that float on water, and duckweeds indeed impose much less control on either growth or stomata [35]. In addition, terrestrial plants curb growth under limiting nitrogen supply in the soil [37,38], whereas duckweeds have a particular propensity for effective nitrogen uptake from the growth medium [39] and an expanded set of genes for nitrogen uptake and amino acid synthesis [34].

### 4.2. Specific Features That May Contribute to Duckweed’s High Shade Tolerance

As noted by Stewart et al. [2], *L. gibba* cultivated under favorable controlled conditions exhibited thin leaves with apparent minimal self-shading.

Concerning foliar pigment composition, principal component analysis revealed that neither of the two *Lemna* species examined here clustered with other fast-growing species, and instead clustered with slow-growing, highly shade-tolerant evergreens, and perennials growing in full sun (pond *L. minor* and *L. gibba* grown under the low PFD) or fell outside either cluster (*L. gibba* grown under the extremely high PFD). Foliar pigment patterns of evergreen and perennial species can be differentiated from those of fast-growing, herbaceous annuals, and biennials by comparatively high total Chl contents with lower levels of VAZ pool carotenoids and β-carotene relative to total Chl, and lower Chl *a*/*b* ratios but comparatively greater levels of lutein relative to Chl in the evergreens and perennials [21]. These features are all consistent with a high light-harvesting capacity associated with comparatively large Chl *b*- and lutein-containing light-harvesting antennae and smaller β-carotene-binding inner antennae [40]. One can thus describe *Lemna* as being unusual in combining fast growth with high shade tolerance. The shade tolerance of duckweeds and their ability to maintain a high growth rate under very low growth PFD may also be due to the fact that floating plants with much-reduced root structures do not need to support a significant proportion of non-photosynthetic tissue, which is challenging in very low light.

Another aspect of duckweed physiology that would support high growth rates in low light, where photosynthetic light-use efficiency must be high, is a rapid return to high photochemical efficiency upon transfer from high to low PFD. This was seen in the present study in the form of a rapid lowering of the rate of thermal energy dissipation upon transfer of high-light-grown *L. gibba* to low light with little to no sustained depression of photosystem II photochemical efficiency or photoinhibition. Notably, such rapid return to a high PSII photochemical efficiency is also seen in shade-tolerant species subsequent to exposure to rapid sunflecks in natural understory settings [3,41] as well as in sun-grown plants of terrestrial Araceae upon return to low light after extended exposure to high light ([42]; see also [43]).

Yet another feature *L. gibba* shares with terrestrial Araceae is maintenance of a similar photosynthetic capacity on an area basis across a wide range of growth PFDs, as reported by Stewart et al. [2] for *L. gibba* grown under PFDs from 100 to 700 µmol photons m^−2^ s^−1^. This trend is also reminiscent of what was reported for *Monstera deliciosa*. When grown under high versus low PFDs, *M. deliciosa* maintained a similar photosynthetic capacity and adjusted its capacity for regulated, photoprotective dissipation of excess absorbed light (not utilized in photochemistry), whereas fast-growing annuals strongly adjusted their photosynthetic capacities with little to no difference in the capacity for photoprotective energy dissipation [30]. The finding of the present study that photosynthetic capacity as well as relative growth rate in fronds grown under 50 µmol photons m^−2^ s^−1^ was similarly high as those of fronds grown over a range of 100 to 700 µmol photons m^−2^ s^−1^ [2] further supports *L. gibba*’s tendency to maintain a similar photosynthetic capacity across a wide range of growth PFDs (see more below on what that means for its high-light tolerance). Concerning *L. gibba*’s shade tolerance, one could speculate that its ability to support a high photosynthetic capacity under low growth PFD may contribute to its high growth rate under very low PFD. It is possible that a high level of the CO_2_-fixing enzyme Rubisco may be associated with duckweed’s propensity to accumulate vegetative storage protein throughout the plant rather than storing protein only in the seed like soybean (duckweed can produce 20× more protein per hectare than soybean [6]). Martindale and Bowes [44] described an unusual propensity of duckweed to accumulate high levels of Rubisco across a range of growth PFDs. While *L. gibba* plants contained more protein on an area basis under 1000 versus 50 µmol photons m^−2^ s^−1^, protein level relative to dry biomass was actually higher under the low growth PFD (biomass was 46% protein) compared to the high growth PFD (biomass was 25% protein on a gram per gram basis). High-quality plant-based protein from duckweeds could thus be produced highly efficiently under low growth PFD.

### 4.3. Features That Likely Contribute to Duckweed’s Tolerance of High Light

Evergreens and perennials often exhibit relatively lower maximal electron transport rates associated with very high fractions (around 90%) of absorbed light allocated to non-photochemical routes as well as very high fractions of the xanthophyll cycle pool converted to zeaxanthin at midday in sunny, but otherwise favorable, habitats [3,16,22,45]. In contrast, annuals and biennials often exhibit relatively higher maximal electron transport rates and relatively lower fractions (around 50%) of absorbed light allocated to non-photochemical routes and fractions of the xanthophyll cycle pool converted to zeaxanthin at midday in the same habitats [3,16,22,45]. In a comparison of the response of terrestrial annual species with the evergreen *M. deliciosa* to a range of growth PFDs, the annuals exhibited pronounced differences in photosynthetic capacity on a leaf area basis with no or only modest differences in photoprotective dissipation of excess excitation energy over a wide range of growth PFDs (with midday peaks of 300 versus 1500 µmol m^−2^ s^−1^), whereas *M. deliciosa* showed no difference in photosynthetic capacity on a leaf area basis but a higher level of both thermal energy dissipation and zeaxanthin content at the higher growth PFD [30]. Duckweed exhibited similar features as *M. deliciosa*, with a pronounced increase in the fraction of absorbed light allocated to energy dissipation via regulated non-photochemical routes and of zeaxanthin accumulation, but no change in photosynthetic capacity, with increased growth PFD. This profoundly greater non-photochemical dissipation of absorbed light in the fronds grown under 1000 µmol photons m^−2^ s^−1^ was apparently highly effective in limiting the build-up of excitation energy (absorbed light not utilized via either photochemistry or non-photochemical routes), as demonstrated in the present study.

At the same time, foliar pigment composition of *Lemna* was distinctive; even shade-grown fronds not containing zeaxanthin exhibited an overall pigment pattern similar to that of sun-grown terrestrial perennials. Furthermore, yellow fronds grown under an extremely high light supply exhibited a much-exaggerated version of this pattern. These features further illustrate that *Lemna* is unusual in combining fast growth with a distinct pigment composition. The low Chl *a* + *b* content, and concomitant high Chl *a*/*b* ratio, in *L gibba* grown under continuous light of 1000 µmol photons m^−2^ s^−1^ is consistent with a strong downregulation of antenna size, which is different from the response of evergreens that, as their name indicates, exhibit limited variation of chlorophyll content. Further support for a small antenna size in *L. gibba* grown under the high PFD comes from the much lower neoxanthin concentration (lowered in proportion of Chl *a* + *b*) and the lower levels of β-carotene and lutein on a frond area basis. The fact that xanthophyll cycle pool, the concentrations of antheraxanthin and zeaxanthin, and the ratio of total xanthophylls to β-carotene were all greater on a frond area basis at the high PFD is presumably due to strong upregulation of zeaxanthin-based photoprotection that may take place not only in pigment-binding protein complexes, but also in the membrane phospholipid bilayer [46].

Since light supply was 20× greater at 1000 versus 50 µmol photons m^−2^ s^−1^ and total dry biomass produced was only just under 3× greater, the biomass produced per mol photons (i.e., the light-use efficiency of biomass production) was dramatically lower at the high PFD. The significant linear relationships between the light-use efficiency of total biomass production and either the fluorescence-derived parameter F_v_′/F_m_′ × q_P_ or the fraction of the xanthophyll cycle pool converted to its de-epoxidized components showed that features associated with primary photosynthetic processes can serve as indicators of duckweed productivity across a range of growth PFDs, irrespective of possible variations of biomass composition with respect to the proportion of, for example, protein, starch, or pigments. Duckweed biomass is particularly valuable with high levels of protein and starch, as previously noted [2]. These relationships may also be as straight-forward in duckweed because this species consists of a one-layer canopy of fronds with minimal non-photosynthetic tissue.

Both F_v_/F_m_ and F_v_′/F_m_′ × q_P_ were also correlated with measures of productivity in rice (see [47]). Prediction of productivity of other systems including whole ecosystems, from parameters associated with primary photosynthetic events is possible but requires consideration of additional features [48,49]. Whereas dark F_v_/F_m_ was shown to be closely correlated with light-use efficiency of photosynthetic electron transport (from O_2_ evolution [50,51]), and F_v_′/F_m_′ × q_P_ is frequently used to estimate photochemical efficiency under illumination [52], these relationships can be tenuous [17] as was also recently discussed by Sipka et al. [53]. Nevertheless, our result that either F_v_′/F_m_′ × q_P_ or xanthophyll cycle pool conversion correlated closely with the light-use efficiency of plant productivity in duckweed is consistent with the assumption that the activity of any additional dissipative processes varies in proportion with the regulated non-photochemical dissipation of excitation energy associated with de-epoxidized xanthophyll cycle components in this species. Future research should examine a possible involvement of alternative photochemical sinks for excitation energy (other than carbon fixation [54]; see also [55,56]) such as oxygen reduction by electron transport, photorespiration, and nitrogen reduction (especially by plastid nitrite reductase). In *Lemna*, nitrogen reduction could be of interest because of the demonstrated enrichment in the duckweed genome of core enzymes in amino acid synthesis [34] and the propensity of duckweed to produce vegetative storage protein.

In the present study, light- and CO_2_-saturated maximal photosynthetic capacity on a frond area or dry mass basis was lower in fronds grown under 1000 µmol photons m^−2^ s^−1^ compared to fronds grown under either 50 µmol photons m^−2^ s^−1^ (this report) or under 100 to 700 µmol photons m^−2^ s^−1^ [2]. This lower maximal photosynthetic capacity did not, however, lead to a lower growth rate in the fronds grown under 1000 versus 50 µmol photons m^−2^ s^−1^, which indicates that at the very high growth PFD of 1000 µmol m^−2^ s^−1^ of continuous light, the somewhat lower photosynthetic capacity was sufficient to support the same high growth rate as at 50 µmol photons m^−2^ s^−1^. The lower photosynthetic capacity on an area or dry mass basis in fronds grown under 1000 µmol photons m^−2^ s^−1^ thus represents an adjustment that allows the plants to maintain a similar growth rate with a lesser photosynthetic capacity and a much lower chlorophyll content in a growth environment with a very high light supply. This feature could also be associated with duckweed accumulating Rubisco levels in excess of what is needed for CO_2_ fixation at a given time. Total Rubisco level may be associated with light- and CO_2_-saturated photosynthetic capacity, but varying proportions of this capacity may be sufficient to support growth under different growth PFDs [44]. The observed extremely high capacity of photosynthetic O_2_ evolution on a Chl basis in fronds grown under 1000 µmol photons m^−2^ s^−1^ of continuous light is consistent with a strong preferential downregulation of antenna size relative to the components of photosynthetic electron transport under this high growth PFD.

Furthermore, *L. gibba* accumulated considerable starch at higher growth PFDs including under 700 µmol photons m^−2^ s^−1^ where no decline in photosynthetic capacity was seen [2], which suggests that the lower photosynthetic capacity in plants grown under 1000 µmol photons m^−2^ s^−1^ may not be due to downregulation associated with starch accumulation. *Lemna*’s propensity for unabated growth and photosynthetic activity over a wide range of growth environments is consistent with the reported reduction of control by water and nutrient level in duckweeds coupled with their genome reduction and associated permanently open stomates and highly effective nutrient acquisition [35].

In a nutshell, *L. gibba* plants grown under continuous high light of 1000 µmol photons m^−2^ s^−1^ effectively counteracted build-up of potentially dangerous excess excitation through a combination of strong downregulation of antenna size (i.e., how much light is absorbed) with strong non-photochemical dissipation of excess absorbed light under the high growth PFD. These photoprotective processes did not interfere with the ability of photosynthetic electron transport to support similar area production and higher dry biomass production in the plants grown under 1000 versus 50 µmol photons m^−2^ s^−1^. This high light tolerance of duckweed is reminiscent to that of the alga *Chlorella ohadii*, which can thrive under a light intensity equivalent to twice that of sunlight [57]. Under such high light conditions, this alga also exhibited unabated fast growth, a high photosynthesis rate, a small antenna size (a constitutive small antenna and an extremely high Chl *a*/*b* ratio of 13:1), and photoprotective energy dissipation that, however, relied on mechanisms other than zeaxanthin-associated non-photochemical energy dissipation [57]. The latter results and those reported here illustrate the existence of photosynthetic systems that grow at high rates under extremely high light and use unique combinations of photoprotective mechanisms. Duckweed is presumably well adapted to a range of natural environments that include predominantly either continuously shaded or high-light-exposed sites (where antenna size modulation should be particularly beneficial) as well as sites with rapidly fluctuating light (where the rapid reversibility of non-photochemical dissipation should be particularly beneficial).

In conclusion, the duckweed *L. gibba* was evidently able to acclimate to very high growth light intensity through a combination of a high growth rate with pronounced starch and protein accumulation, decreased light absorption (presumably by downregulation of antenna size), pronounced non-photochemical dissipation of excess light associated with zeaxanthin as well as other forms of photoprotection provided by the xanthophyll lutein (that can remove excitation energy from Chl in the triplet state [58]), β-carotene (that can contribute to the photoprotection of photosystem I [59]), and α-tocopherol (that can scavenge singlet oxygen and lipid peroxy radicals [60,61]). The greater levels of especially zeaxanthin, α-tocopherol, lutein, and to some extent β-carotene relative to Chl under high growth PFD are consistent with an enhanced need for photoprotection on part of the plant. From the standpoint of human/animal nutrition, however, production of micronutrients per area or as a percent of biomass matters most. Whereas zeaxanthin production required high light irrespective of reference basis, α-tocopherol (vitamin E), lutein, β-carotene (provitamin A), and protein levels as a percent of biomass were all lower under the high growth PFD. Therefore, a mixed lighting protocol with mainly low background PFD, supplemented with brief exposures to high light (see, e.g., [62]) might be attractive to produce high-quality nutrition for the consumer.

## Figures and Tables

**Table 1:** Levels of carotenoids and α-tocopherol per frond area and dry mass (DM) in fronds of *Lemna gibba* grown under PFDs of 50 or 1000 µmol m^−2^ s^−1^.

Units	Compound(s)	Growth PFD (µmol m^−2^ s^−1^)		*p*
		50	1000	
(µmol m^−2^)	Zeaxanthin	1 ± 0	35 ± 2	***
	Antheraxanthin	1 ± 0	15 ± 1	***
	Violaxanthin	22 ± 1	3 ± 0	***
	Lutein	43 ± 2	26 ± 2	***
	Neoxanthin	13 ± 0	3 ± 0	***
	β-Carotene	19 ± 3	9 ± 1	**
	Z + A	2 ± 1	50 ± 3	***
	V + A + Z	24 ± 1	54 ± 3	***
	α-Tocopherol	5 ± 1	5 ± 1	*n.s.*
(mmol g^−1^ DM)	Zeaxanthin	30 ± 12	843 ± 60	***
	Antheraxanthin	57 ± 26	362 ± 23	***
	Violaxanthin	1156 ± 56	77 ± 11	***
	Lutein	2214 ± 124	627 ± 37	***
	Neoxanthin	692 ± 20	78 ± 10	***
	β-Carotene	995 ± 157	212 ± 13	***
	Z + A	88 ± 38	1205 ± 64	***
	V + A + Z	1255 ± 55	1282 ± 62	*n.s.*
	Carotenoids	5192 ± 220	2198 ± 113	***
	α-Tocopherol	241 ± 38	113 ± 18	**

Mean values ± standard deviations; *n* = 3 or 4. Significant differences between growth PFDs are denoted by asterisks; ** = *p* < 0.01; *** = *p* < 0.001; *n.s.* = not significantly different. A = antheraxanthin, V = violaxanthin, Z = zeaxanthin.

**Table 2:** Levels of carotenoids and α-tocopherol relative to chlorophylls (Chl) or other carotenoids in fronds of *Lemna gibba* grown under PFDs of 50 or 1000 µmol m^−2^ s^−1^.

Units	Compound(s)	Growth PFD (µmol m^−2^ s^−1^)		*p*
		50	1000	
(mmol mol^−1^ Chl *a* + *b*)	Lutein	160 ± 8	421 ± 25	***
	Neoxanthin	50 ± 2	52 ± 3	*n.s.*
	β-Carotene	72 ± 11	142 ± 5	***
	Z + A	6 ± 3	811 ± 57	**
	V + A + Z	90 ± 5	862 ± 52	***
	Carotenoids	371 ± 14	1478 ± 75	**
	α-Tocopherol	17 ± 3	73 ± 12	**
(mmol mol^−1^ Chl *b*)	Zeaxanthin	9 ± 4	3372 ± 301	**
	Antheraxanthin	17 ± 8	1445 ± 61	***
	Violaxanthin	330 ± 14	307 ± 44	*n.s.*
	Lutein	632 ± 24	2504 ± 125	***
	Neoxanthin	198 ± 10	308 ± 12	***
	β-Carotene	284 ± 47	847 ± 52	***
	Z + A	25 ± 12	4818 ± 306	**
	V + A + Z	356 ± 20	5125 ± 289	***
	Carotenoids	1470 ± 61	8784 ± 416	***
	α-Tocopherol	69 ± 11	446 ± 72	***
(mol mol^−1^)	Xanthophylls/β-Carotene	4.25 ± 0.71	9.39 ± 0.67	***
	Zeaxanthin/Lutein	0.01 ± 0.01	1.35 ± 0.05	***
	(V + A + Z)/Lutein	0.56 ± 0.03	2.05 ± 0.02	***
(% of V + A + Z)	Zeaxanthin	2 ± 1	66 ± 3	***
	Z + A	7 ± 3	94 ± 1	***

Mean values ± standard deviations; *n* = 3 or 4. Significant differences between growth PFDs are denoted by asterisks; ** = *p* < 0.01; *** = *p* < 0.001; *n.s.* = not significantly different. A = antheraxanthin, V = violaxanthin, Z = zeaxanthin.

## Data Availability

The data presented in this study are available from the corresponding author upon reasonable request.

## Supplementary Materials

The following are available online at https://www.mdpi.com/article/10.3390/cells10061481/s1, Table S1: Source, species, and grouping of data used for principal component analysis of pigment composition (see Figure 10), as well as scores for all eight of the resulting principal components; Table S2: Loading values of the eight pigment parameters used for principal component analysis (see Figure 10) for the eight resulting principal components; Table S3: Correlation matrix for the eight variables used in the principal component analysis of pigment composition (see Figure 10).
